# A Hybrid Swarm Intelligence Algorithm for Intrusion Detection Using Significant Features

**DOI:** 10.1155/2015/574589

**Published:** 2015-06-22

**Authors:** P. Amudha, S. Karthik, S. Sivakumari

**Affiliations:** ^1^Department of CSE, Avinashilingam Institute for Home Science and Higher Education for Women, Coimbatore 641 108, India; ^2^Department of CSE, SNS College of Technology, Coimbatore 641 035, India

## Abstract

Intrusion detection has become a main part of network security due to the huge number of attacks which affects the computers. This is due to the extensive growth of internet connectivity and accessibility to information systems worldwide. To deal with this problem, in this paper a hybrid algorithm is proposed to integrate Modified Artificial Bee Colony (MABC) with Enhanced Particle Swarm Optimization (EPSO) to predict the intrusion detection problem. The algorithms are combined together to find out better optimization results and the classification accuracies are obtained by 10-fold cross-validation method. The purpose of this paper is to select the most relevant features that can represent the pattern of the network traffic and test its effect on the success of the proposed hybrid classification algorithm. To investigate the performance of the proposed method, intrusion detection KDDCup'99 benchmark dataset from the UCI Machine Learning repository is used. The performance of the proposed method is compared with the other machine learning algorithms and found to be significantly different.

## 1. Introduction

Due to the tremendous growth in the field of information technology, one of the significant challenging issues is network security. Hence, intrusion detection system (IDS) which is an indispensable component of the network needs to be secured. The traditional IDS is unable to handle newly arising attacks. The main goal of IDSs is to identify and distinguish the normal and abnormal network connections in an accurate and quick manner which is considered as one of the main issues in intrusion detection system because of the large amount of attributes or features. To study about this aspect, data mining based network intrusion detection is widely used to identify how and where the intrusions occur. Related to achieving real-time intrusion detection, researchers have investigated several methods of performing feature selection. Reducing the number of features by selecting the important features is critical to improve the accuracy and speed of classification algorithms. Hence, selecting the differentiating features and developing the best classifier model in terms of high accuracy and detection rates are the main focus of this work.

The research on machine learning or data mining considers the intrusion detection as a classification problem, implementing algorithms such as Naïve Bayes, genetic algorithm, neural networks, Support Vector Machine, decision trees. In order to improve the accuracy of an individual classifier, popular approach is to combine the classifiers. Recently, application of swarm intelligence technique for intrusion detection has gained prominence among the research community [1]. Swarm intelligence can be a measure presenting the communal behaviour of social insect colonies or other animal societies to implement algorithms [2]. The potential of swarm intelligence makes it a perfect candidate for IDS, which needs to distinguish normal and abnormal behaviors from large amount of data.

The main objective of this work is (1) to select important features using two feature selection methods, namely, single feature selection method and random feature selection method and (2) to propose a hybrid optimization algorithm based on Artificial Bee Colony (ABC) and Particle Swarm Optimization (PSO) algorithms for classifying intrusion detection dataset. The studies on ABC and PSO indicate that ABC has powerful global search ability but poor local search ability [3], while the PSO has powerful local search ability but poor global search ability [4]. In order to provide a powerful global search capability and local search capability, in this paper a hybridized model called MABC-EPSO is proposed which brings the two algorithms together so that the computation process may benefit from both advantages. In this hybrid algorithm, the local search and global search abilities are balanced to obtain more quality results. KDDCUP'99 intrusion detection dataset developed by MIT Lincoln Laboratory is used for experiments to find the accuracy of the proposed hybrid approach.

The rest of this paper is organized as follows.  Section 2 provides an overview of related work.  Section 3 presents the principles of PSO and ABC.  Section 4 describes the methodology, dataset description and preprocessing, proposed feature selection methods, and hybrid approach.  Section 5 gives performance metrics, experimental results, and discussions. Finally, conclusion is given in Section 6.

## 2. Related Work

Being related to achieving real-time intrusion detection, researchers have investigated several methods of performing feature selection. Kohavi and John [4] described the feature subset selection problem in supervised learning, which involves identifying the relevant or useful features in a dataset and giving only that subset to the learning algorithm. The real-life intrusion detection dataset contains redundant features or insignificant features. The redundant features make it harder to detect possible intrusion patterns [5]. With the increasing applications of classification algorithms and feature selection methods for intrusion detection dataset, a comprehensive list of a few such literatures is given in [6–23].

Machine learning algorithms such as neural networks [9], fuzzy clustering [14] have been applied to IDS to construct good detection model. Support vector machine (SVM) [24] has become a popular research method in intrusion detection due to its good generalization performance and the sparse representation of solution. Satpute et al. [25] enhanced the performance of intrusion detection system by combining PSO and its variants with machine learning techniques for the detection of anomaly in network intrusion detection system. Chung and Wahid [26] proposed a novel simplified swarm optimization (SSO) algorithm as a rule based classifier and for feature selection for classifying audio data. The algorithm is more flexible and cost-effective to solve complex computing environments. Revathi and Malathi [10, 11] proposed hybrid simplified swarm optimization to preprocess the data and compared the proposed approach with a new hybridized approach, PSO with Random Forest, and found that the proposed method provided high detection rate and optimal solution.

Karaboga and Basturk [27] proposed Artificial Bee Colony (ABC) algorithm based on a particular intelligent behaviour of honeybee swarms. By understanding the basic behaviour characteristics of foragers, ABC algorithm was developed and was compared with that of differential evolution, Particle Swarm Optimization, and evolutionary algorithm for multidimensional and multimodal numeric problems. Karaboga and Akay [28] proposed ABC algorithm for anomaly-based network intrusion detection system to optimize the solution. The proposed method was classified into four stages such as parameterization, training stage, testing stage, and detection stage. D. D. Kumar and B. Kumar [29] applied ABC algorithm for anomaly-based IDS and used feature selection techniques to reduce the number of features used for detection and classification. Mustafa ServetKiran and MesutGunduz [30] proposed a hybridization of PSO and ABC for different continuous optimization problems in which the information between particle swarm and bee colony helps in increasing global and local search abilities of the hybrid approach.

## 3. Theoretical Background

The following subsections provide the necessary background to understand the problem.

### 3.1. Particle Swarm Optimization

Particle Swarm Optimization (PSO) is one of the popular heuristic technique which has been successfully applied in many different application areas, but, however, it suffers from premature convergence especially in high dimension, multimodal problems.

The algorithm of the standard PSO is as follows.

1. Initialize a population of particles with randomly chosen positions and velocities.
2. Calculate the fitness value of each particle in the population.
3. If the fitness value of the particle *i* is better than its* pbest* value, then set the fitness value as a new* pbest* of particle *i*.
4. If* pbest* is updated and it is better than the current* gbest*, then set* gbest* to the current* pbest* value of particle *i*.
5. Update particle's velocity and position according to (1) and (2).
6. If the best fitness value or the maximum generation is met, then stop the process; otherwise, repeat the process from step  2.

In PSO, a swarm consists of *N* particles in a* D*-dimensional searching space. The *i*th particle is represented as *X*_*i*_ = (*x*_*i*1_, *x*_*i*2_,…, *x*_*id*_). The best previous position* pbest* of any particle is *P*_*i*_ = (*p*_*i*1_, *p*_*i*2_,…, *p*_*id*_) and the velocity for particle *i* is *V*_*i*_ = (*v*_*i*1_, *v*_*i*2_,…, *v*_*id*_). The global best particle in the whole swarm is denoted by *P*_*g*_ and it represents the fittest particle [31]. During each iteration, each particle updates its velocity according to the following equation:

(1) vidt=vidt−1+c1·rand1⁡·pid−xidt−1+c2·rand2⁡·pgd−xidt−1,

where *c*_1_ and *c*_2_ denote the acceleration coefficients, *d* = 1,2,…, *D*, and rand_1_ and rand_2_ are random numbers uniformly distributed within [0,1].

Each particle then moves to a new potential position as in the following equation:

(2) $$\begin{array}{c} x_{id}^{t}=x_{id}^{t-1}+v_{id}^{t},\;d=1,2,\ldots ,D. \end{array}$$

### 3.2. Artificial Bee Colony

The Artificial Bee Colony (ABC) algorithm is an optimization algorithm based on the intelligent foraging behaviour of honey bee swarm, proposed by Karaboga and Basturk [27]. The Artificial Bee Colony comprises of three groups: scout bees, onlooker bees, and employed bees. The bee, which carries out random search, is known as scout bee. The bee which visits the food source is an employed bee. The bee, which waits on the dance region is an onlooker bee and the onlooker bee with scout is also called unemployed bee. The employed and unemployed bees search for the good food sources around the hive. The employed bees share the stored food source information with onlooker bees. The amount of food sources is equal to the amount of employed bees and also is equal to the number of onlooker bees. The solutions of the employed bees which cannot be enhanced by a fixed number of bounds become scouts and their solutions are abandoned [28]. In the context of optimization, the amount of food sources in ABC algorithm represents the number of solutions in the population. The point of a good food source indicates the location of a promising solution to the optimization problem [27].

The four main phases of ABC algorithm are as follows.

*Initialization Phase*. The scout bees randomly generate the population size (SN) of food sources. The input vector *x*_*m*_ which contains *D* variables represents food source where *D* represents the searching space dimension of the objective function to be optimized. Using (3), initial sources of food are produced randomly

(3) $$\begin{array}{c} x_{m}=l_{i}+\mathrm{rand}\left(\;0,1\;\right)*\left(\;u_{i}-l_{i}\;\right), \end{array}$$

where *u*_*i*_ and *l*_*i*_ are the upper and lower bounds of the solution space of objective function and rand(0,1) is a random number within the range [0, 1].

*Employed Bee Phase*. The employed bee finds a new food source within the region of the food source. The employed bees reminisce higher quantity of food source and share it with onlooker bees. Equation (4) determines the neighbour food source *v*_*mi*_ and is calculated by

(4) $$\begin{array}{c} v_{mi}=x_{mi}+\phi_{mi}\left(\;x_{mi}-x_{ki}\;\right), \end{array}$$

where *i* is a randomly selected parameter index, *x*_*k*_ is a randomly selected food source, and *ϕ*_*mi*_ is a random number within the range [−1,1]. Suitable tuning on specific problems can be made using this parameter range. The fitness of food sources, which is needed to find the global, optimal solution, is calculated by (5). And a greedy selection method is used between *x*_*m*_ and *v*_*m*_

(5) $$\begin{array}{c} {fit}_{i}=\left\{\begin{array}{cc} \frac{1}{f_{i}+1}, & f_{i}\geq 0\\ 1+\left|f_{i}\right|, & f_{i}<0, \end{array}\right. \end{array}$$

where *f*_*i*_ represents the objective value of *i*th solution.

*Onlooker Bee Phase*. Onlooker bees examine the effectiveness of food sources by observing the waggle dance in the dance region and then randomly select a rich food source. Then, the bees perform a random search in the neighbourhood area of food source using (4). The quantity of a food source is evaluated by its profitability *P*_*i*_ using the following equation:

(6) $$\begin{array}{c} p_{i}=\frac{{\text{fit}}_{i}}{\sum_{n=1}^{SN}{\text{fit}}_{n}}, \end{array}$$

where fit_*i*_ denotes the fitness of the solution represented by food source *i* and SN denotes the total number of food sources which is equal to number of employed bees.

*Scout Phase*. If the effectiveness of food source cannot be improved by the fixed number of trials, then the scout bees remove the solutions and randomly search for new solutions by using (3) [29].

The pseudocode of the ABC algorithm is given in Algorithm 1.

## 4. Methodology

### 4.1. Research Framework

In this study, the framework of the proposed work is given as follows.

- Data preprocessing: it prepared the data for classification and removed unused features and duplicate instances.
- Feature selection: it determined the feature subset using SFSM and RFSM methods that contribute to the classification.
- Hybrid classification: it performed classification using MABC-EPSO algorithm to enhance the classification accuracy for the KDDCUP'99 dataset.

The objective of this study is to help the network administrator in preprocessing the network data using feature selection methods and to perform classification using hybrid algorithm which aims to fit a classifier model to the prescribed data.

### 4.2. Data Source and Dataset Description

In this section, we provide brief description of KDDCup'99 dataset [30] which is derived from UCI Machine Learning Repository [31]. In 1998, DARPA intrusion detection evaluation program, to perform a comparison of various intrusion detection methods, a simulated environment, was set up by the MIT Lincoln Lab to obtain raw TCP/IP dump data for a local-area network (LAN). The functioning of the environment was like a real one, which included both background network traffic and wide variety of attacks. A version of 1998 DARPA dataset, KDDCup'99, is now widely accepted as a standard benchmark dataset and received much attention in the research community of intrusion detection. The main motivation of using KDDCup'99 Dataset is to show that the proposed method has the advantage of becoming an efficient classification algorithm when applied to the intrusion detection system. In this paper, 10% KDD Cup'99 dataset is used for experimentation. The distribution of connection types and sample size in 10% KDDCUP dataset is shown in Tables 1 and 2. The feature information of 10% KDDCUP dataset is shown in Table 3. The dataset consists of one type of normal data and 22 different attack types categorized into 4 classes, namely, denial of service (DoS), Probe, user-to-root (U2R), and remote-to-login (R2L).

### 4.3. Data Preprocessing

**Figure 1:**
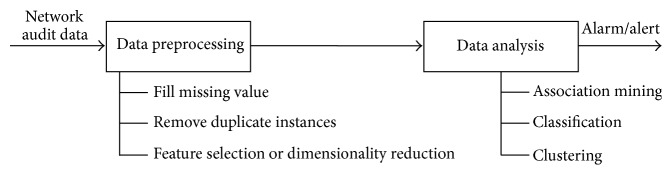
Data preprocessing.

Data preprocessing is the time-consuming task which prepares the data for subsequent analysis as per the requirement for intrusion detection system model. The main aim of data preprocessing is to transform the raw network data into suitable form for further analysis.  Figure 1 illustrates the steps involved in data processing and how raw input data are processed for further statistical measures.

Various statistical analyses such as feature selection, dimensionality reduction, and normalization are essential to select significant features from the dataset. If the dataset contains duplicate instances, then the classification algorithms consume more time and also provide inefficient results. To achieve more accurate and efficient model, duplication elimination is needed. The main deficiency in this dataset is the large number of redundant instances. This large amount of duplicate instances will make learning algorithms be partial towards the frequently occurring instances and will inhibit it from learning infrequent instances which are generally more unsafe to networks. Also, the existence of these duplicate instances will cause the evaluation results to be biased by the methods which have better detection rates on the frequently occurring instances [32]. Eliminating duplicate instances helps in reducing false-positive rate for intrusion detection. Hence, duplicate instances are removed, so the classifiers will not be partial towards more frequently occurring instances. The details of instances in the dataset are shown in Table 4. After preprocessing, selected random sample of 10% normal data and 10% Neptune attack in DoS class and four new sets of data are generated with the normal class and four categories of attack [33]. Moreover, irrelevant and redundant attributes of intrusion detection dataset may lead to complex intrusion detection model and reduce detection accuracy.

### 4.4. Feature Selection

Feature selection is an important data processing process. As the dataset is large, it is essential to remove the insignificant features, in order to distinguish normal traffic or intrusions in a well-timed manner. In this paper, feature subsets are formed based on single feature method (SFSM), random feature selection method (RFSM) and compared the two techniques. The proposed methods reduce the features in the datasets which aim to improve accuracy rate, reduce processing time, and improve efficiency for intrusion detection.

#### 4.4.1. Single Feature Selection Method

Single feature method (SFSM) uses the one-dimensional feature vector. In the first iteration, it considers only the first attribute and is evaluated for calculating the accuracy using the Support Vector Machine classifier. In the second iteration, it considers only the corresponding attribute for evaluation. The process is repeated until all 41 features are evaluated. After calculating the entire feature's efficiency, it is sorted and vital features are selected, whose accuracy and detection rate are acc_threshold and dr_threshold values, respectively. The pseudocode of single feature selection algorithm is given in Algorithm 2.

#### 4.4.2. Random Feature Selection Method

In this method, the features are removed randomly and evaluated using the classifier. In the first iteration, all the features are evaluated using SVM classifier, and then by deleting one feature, update the dataset, using the classifier efficiency. The importance of the provided feature is calculated. In the second iteration, another feature is removed randomly from the dataset and updated. The process is repeated until only one feature is left. After calculating the entire feature's efficiency, it is sorted in descending order of its accuracy. If the accuracy and detection rate are greater than the threshold value (accuracy and detection rate obtained using all features), then select those features as vital features. The pseudocode of the random feature selection algorithm is given in Algorithm 3.

Tables 5 and 6 show the feature subsets identified using the two feature selection methods and size of the subsets identified as a percentage of the full feature set.

### 4.5. Hybrid Classification Approach

Artificial intelligence and machine learning techniques were used to build different IDSs, but they have shown limitations in achieving high detection accuracy and fast processing time. Computational intelligence techniques, known for their ability to adapt and to exhibit fault tolerance, high computational speed, and resilience against noisy information, compensate for the limitations of these approaches [1]. Our aim is to increase the level of performance of intrusion detection of the most used classification techniques nowadays by using optimization methods like PSO and ABC. This work develops an algorithm that combines the logic of both ABC and PSO to produce a high performance IDS and their combination has the advantage of providing a more reliable solution to today's data intensive computing processes.

**Figure 2:**
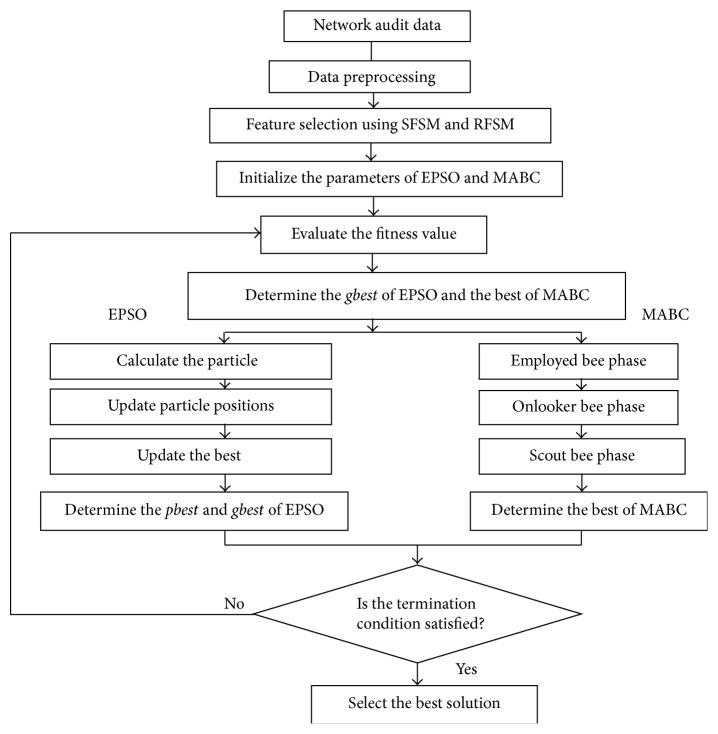
Flowchart of the proposed hybrid MABC-EPSO model.

Artificial Bee Colony algorithm is a newly proposed optimization algorithm and is becoming a hot topic in computational intelligence nowadays. Because its high probability of avoiding the local optima, it can make up the disadvantage of Particle Swarm Optimization algorithm. Moreover, Particle Swarm Optimization Algorithm can help us to find out the optimal solution more easily. In such circumstances, we bring the two algorithms together so that the computation process may benefit from both of the advantages. The flowchart of the proposed hybrid MABC-EPSO is given in Figure 2.

In this hybrid model, the colony is divided into two parts: one possesses the swarm intelligence of Artificial Bee Colony and the other one is the particle swarm intelligence. Assuming that there is cooperation between the two parts, in each iteration, one part which finds out the better solution will share its achievement with the other part. The inferior solution will be replaced by the better solution and will be substituted in the next iteration. The process of MABC-EPSO is as follows.

**Step 1 (initialization of parameters).**

Set the number of individuals of the swarm; set the maximum circle index of the algorithm; set the search range of the solution; set the other constants needed in both ABC and PSO.

**Step 2 (initialization of the colony).**

Generate a colony with a specific number of individuals. Bee colony is divided into two categories, employed foragers and unemployed foragers, according to each individual's fitness value; on the other hand, as a particle swarm, calculate the fitness value of each particle and take the best location as the global best location.

**Step 3 .**

In bee colony, to evaluate the fitness value of each solution, an employee bee is assigned using (5). The employee bee selects a new candidate solution from the nearby food sources and then uses greedy selection method by calculating the Rastrigin function as follows:

(7) $$\begin{array}{c} \mathrm{Min}f\left(\;x\;\right)=10n+\overset{n}{\underset{i=1}{\sum }}\left[\;x_{i}^{2}-10\cos\left(\;2\pi x_{i}\;\right)\;\right]. \end{array}$$

A multimodal function is said to contain more than one local optimum. A function of variables is separable if it can be modified as a sum of functions of just one variable [34]. The dimensionality of the search space is another significant factor in the complexity of the problem. The challenge involved in finding optimal solutions to this function is that, on the way towards the global optimum, an optimization problem can be easily confined in a local optimum. Hence, the classical benchmark function Rastrigin [34] is implemented using Artificial Bee Colony algorithm and named as Modified Artificial Bee Colony (MABC) algorithm. In (1) *f*_*i*_ is Rastrigin function whose value is 0 at its global minimum (0,0,…, 0). This function is chosen, because it is considered to be one of the best test functions for finding the global minimum. Initialization range for the function is [−15, 15]. This function is with cosine modulation to produce many local minima. Thus, the function is multimodal.

**Step 4 .**

If the fitness value is larger than the earlier one, the bee remembers the new point and forgets the previous one; otherwise, it keeps the previous solution. Based on the shared information by employee bees, an onlooker bee calculates the shared fitness value and selects a food source with a probability value computed as in (6).

**Step 5 .**

An onlooker bee constructs a new solution selected among the neighbors of a previous solution. It also checks the fitness value and if this value is better than the previous one, it will substitute the old one with the new position; otherwise, it evokes the old position. The objective of scout bees is to determine new random food sources to substitute the solutions that cannot be enhanced after reaching the “limit” value. In order to obtain the best optimized solution, the algorithm goes through a predefined number of cycles (MCN). After all the choices have been made, the best solution generated in that iteration is called MABC_best_.

**Step 6 .**

As there is a large effect of initial velocity in the balancing of exploration and exploitation process of swarm, in this proposed Enhanced Particle Swarm Optimization (EPSO) algorithm, inertia weight (*ω*) [35] is used to control the velocity and hence the velocity update equation (8) becomes as follows:

(8) vidt=ω·vidt−1+c1·rand1·pid−xidt−1+c2·rand2⁡·pgd−xidt−1.

A small inertia weight facilitates a local search, whereas a large inertia weight facilitates a global search. In the EPSO algorithm, linear decreasing inertia weight [36] as in (9) is used to enhance the efficiency and performance of PSO. It is found experimentally that inertia weight from 0.9 to 0.4 provides the optimal results

(9) $$\begin{array}{c} w_{k}=w_{max}-\frac{w_{max}-w_{min}}{{\text{iter}}_{max}}\times k. \end{array}$$

In particle swarm, after the comparison among the solutions that each particle has experienced and the comparison among the solutions that all the particles have ever experienced, the best location in that iteration is called EPSO_best_.

**Step 7 .**

The minimum of the value MABC_best_ and EPSO_best_ is called Best and is defined as

(10) $$\begin{array}{c} \text{Best}=\left\{\begin{array}{cc} {\text{EPSO}}_{\text{best}} & \text{if }{\text{EPSO}}_{\text{best}}\leq {\text{MABC}}_{\text{best}}\\ {\text{MABC}}_{\text{best}} & \text{if }{\text{MABC}}_{\text{best}}\leq {\text{EPSO}}_{\text{best}}. \end{array}\right. \end{array}$$

**Step 8 .**

If the termination condition is satisfied, then end the process and report the best solution. Otherwise, return to Step 2.

*Parameter Settings*. The algorithms are evaluated using the two feature sets selected by SFSM and RFSM. In ABC algorithm, the parameters set are bee colony size: 40, MCN: 500, and limit: 5. In EPSO algorithm, the inertia weight *ω* in (11) varies from 0.9 to 0.7 linearly with the iterations. Also, the acceleration coefficients *c*_1_ and *c*_2_ are set as 2. The upper and lower bounds for *v*(*v*_min_, *v*_max_) are set as the maximum upper and lower bounds of *x*

(11) vidt=ωvidt−1+c1rand⁡0,1pid−xidt−1+c2rand⁡0,1pgd−xidt−1.

## 5. Experimental Work

This section provides the performance metrics that are used to assess the efficiency of the proposed approach. It also presents and analyzes the experimental results of hybrid approach and compares it with the other classifiers.

### 5.1. Performance Metrics

The performance metrics like accuracy, sensitivity, specificity, false alarm rate, and training time are recorded for the intrusion detection dataset on applying the proposed MABC-PSO classification algorithm. Generally, sensitivity and specificity are the statistical measures used to carry out the performance of classification algorithms. Hence, sensitivity and specificity are chosen to be the parametric indices for carrying out the classification task. In intrusion detection problem, sensitivity can also be called detection rate. The number of instances predicted correctly or incorrectly by a classification model is summarized in a confusion matrix and is shown in Table 7.

The classification accuracy is the percentage of the overall number of connections correctly classified

(12) $$\begin{array}{c} \text{Classification accuracy}=\frac{\left(TP+TN\right)}{\left(TP+TN+FP+FN\right)}. \end{array}$$

Sensitivity (True Positive Fraction) is the percentage of the number of attack connections correctly classified in the testing dataset

(13) $$\begin{array}{c} \text{Sensitivity}=\frac{TP}{\left(TP+FN\right)}. \end{array}$$

Specificity (True Negative Fraction) is the percentage of the number of normal connections correctly classified in the testing dataset

(14) $$\begin{array}{c} \text{Specificity}=\frac{TN}{\left(TP+FN\right)}. \end{array}$$

False alarm rate (FAR) is the percentage of the number of normal connections incorrectly classified in the testing and training dataset

(15) $$\begin{array}{c} \text{False Alarm Rate (FAR)}=\frac{FP}{\left(TN+FP\right)}. \end{array}$$

Cross-validation is a technique for assessing how the results of a statistical analysis will generalize to an independent dataset. It is the standard way of measuring the accuracy of a learning scheme and it is used to estimate how accurately a predictive model will perform in practice. In this work, 10-fold cross-validation method is used for improving the classifier reliability. In 10-fold cross-validation, the original data is divided randomly into 10 parts. During each run, one of the partitions is chosen for testing, while the remaining nine-tenths are used for training. This process is repeated 10 times so that each partition is used for training exactly once. The average of the results from the 10-fold gives the test accuracy of the algorithm [37].

### 5.2. Results and Discussions

The main motivation is to show that the proposed hybrid method has the advantage of becoming an efficient classification algorithm based on ABC and PSO. To further prove the robustness of the proposed method, other popular machine learning algorithms [38] such as Naives Bayes (NB) which is a statistical classifier; decision tree (j4.8); radial basis function (RBF) network; Support Vector Machine (SVM) that is based on the statistical learning theory and basic ABC are tested on KDDCup'99 dataset. For each classification algorithm, their default control parameters are used. In Table 8, the results are reported for accuracy rate obtained by various classification algorithms using different feature selection methods.

**Figure 3:**
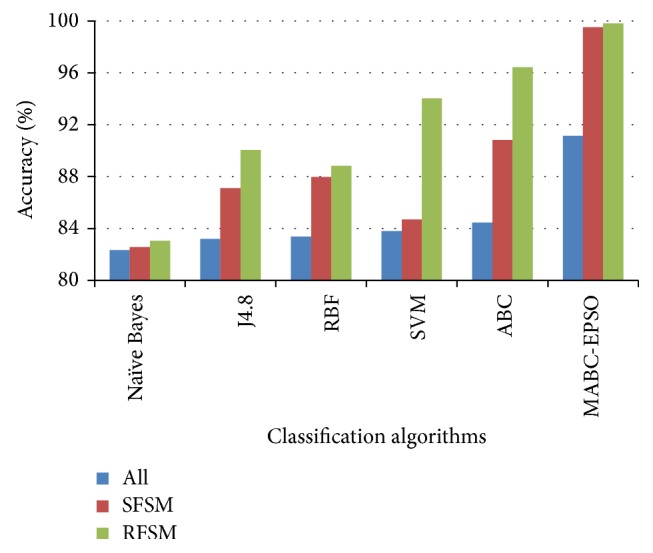
Accuracy comparison of classifiers for DoS dataset.

**Figure 4 fig4:**
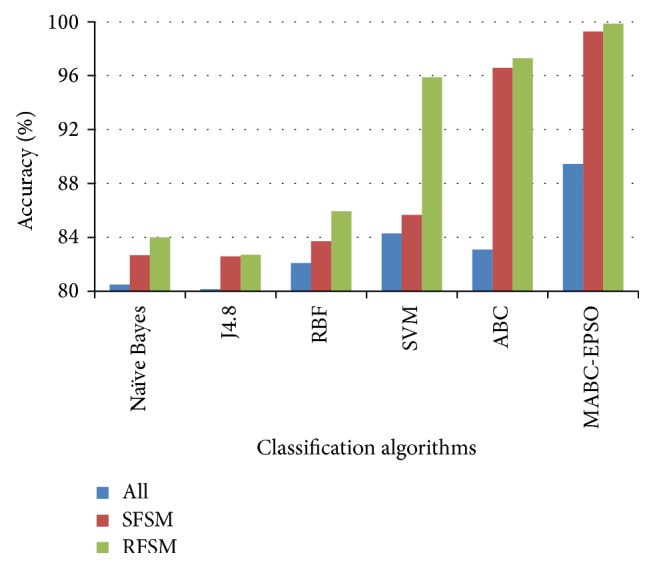
Accuracy comparison of classifiers for probe dataset.

**Figure 5 fig5:**
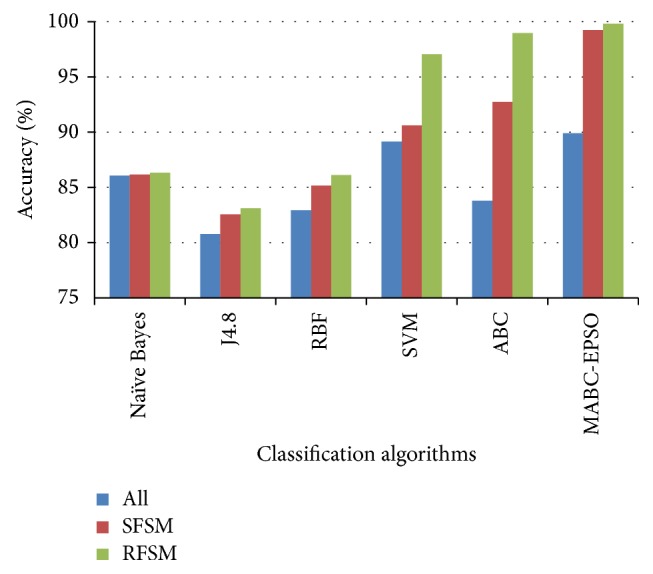
Accuracy comparison of classifiers for R2L dataset.

The performance comparison of the classifiers on accuracy rate is given in Figures 3–6. The results show that, on classifiying the dataset with all features, the average accuracy rate of 85.5%, 84.5%, and 88.59% is obtained for SVM, ABC, and proposed hybrid approaches. When SFSM is applied, accuracy rate of ABC and proposed MABC-EPSO is increased significantly to 94.36% and 99.32%. The highest accuracy (99.82%) is reported when the proposed MABC-EPSO with random feature selection method is employed. It is also observed that on applying random feature selection method, the accuracy of SVM and ABC is increased to 95.71% and 97.92%. The accuracy rate of NB, j4.8, and RBF classifiers is comparatively high with RFSM method compared to SFSM and full feature set.

In order to test the significance of the differences among classifiers, six classification algorithms previously mentioned over four datasets are considered and performed experiments using Friedman test and ANOVA. Tables 9 and 10 depict the classification accuracy using two feature selection methods and their ranks computed through Friedman test (ranking is given in parenthesis). The null hypothesis states that all the classifiers perform in the same way and hence their ranks should be equal. The Friedman test ranked the algorithms for each dataset, with the best performing algorithm getting the rank of 1, the second best algorithm getting the rank 2. As seen in Table 9, MABC-EPSO is the best performing algorithm, whereas Naïve Bayes is the least performing algorithm and Table 10 shows that MABC-EPSO is the best performing algorithm, whereas Naïve Bayes and j4.8 are the least performing algorithms. Friedman statistic *χ* ^2^ = 15.716 and *F*_*F*_ = 11.005 for SFSM and *χ* ^2^ = 15.712 and *F*_*F*_ = 10.992 for RFSM are computed. Having four datasets and six classification algorithms, distribution of *F*_*F*_ is based on *F* distribution with 6 − 1 = 5 and (6 − 1)*∗*(4 − 1) = 15 degrees of freedom. The critical value of *F*(5,15) for *α* = 0.05 is 2.9013 and *P* value < 0.05. So, we reject the null hypothesis, and the differences among classifiers are significant.

The means of several groups by estimating the variances among groups and within a group are compared using the ANOVA test. Here, the null hypothesis which is set as all population means are equal is tested. Also *P* value and the value of *F* are computed. If the null hypothesis is rejected, Tukey's post hoc analysis method is applied to perform a multiple comparison which tests all means pairwise, to determine which ones are significantly different.  Table 11 shows the results determined by ANOVA. In SFSM method, the ANOVA test rejected the null hypothesis, as calculated *F*(5,18) = 31.895 is greater than* F*-critical (2.773) for the significance level of 5%. Tukey's post hoc test is performed which states that significantly there are differences among MABC-EPSO and ABC with other classifiers but not among NB, j4.8, RBF, and SVM. Also, there are significant differences between ABC and MABC-EPSO; so ABC and MABC-EPSO are the best classifiers in this case. In RFSM method, there were statistically significant differences between algorithms and hence null hypothesis was rejected, as the calculated *F*(5,18) = 48.547 is greater than* F*-critical (2.773) for the significance level of 5%. Tukey's posthoc test is performed and it reveals that there is a statistically significant difference among SVM, ABC, and MABC-EPSO with other classifiers but not among NB, j4.8, and RBF. However, there is no statistically significant difference between the ABC and MABC-EPSO algorithms.

**Figure 7:**
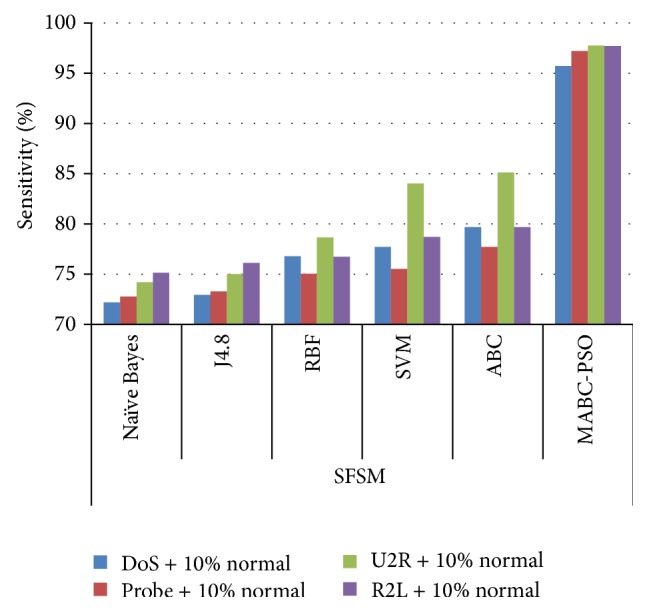
Comparison on sensitivity using SFSM method.

**Figure 8 fig8:**
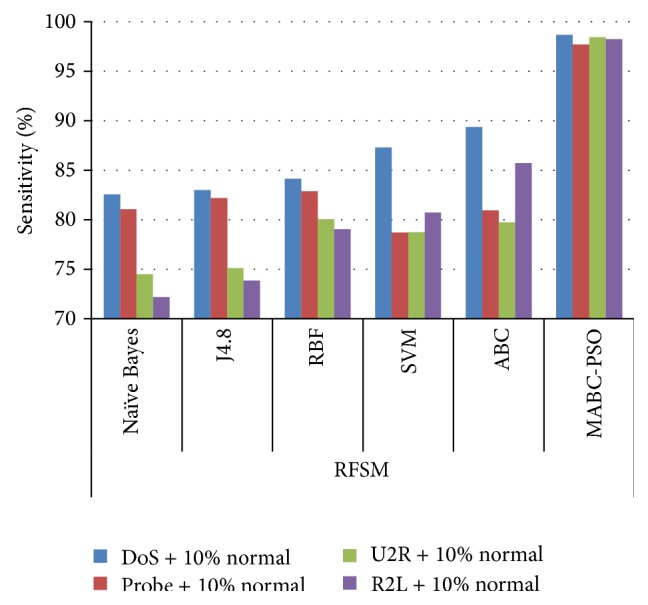
Comparison on sensitivity using RFSM method.

**Figure 9 fig9:**
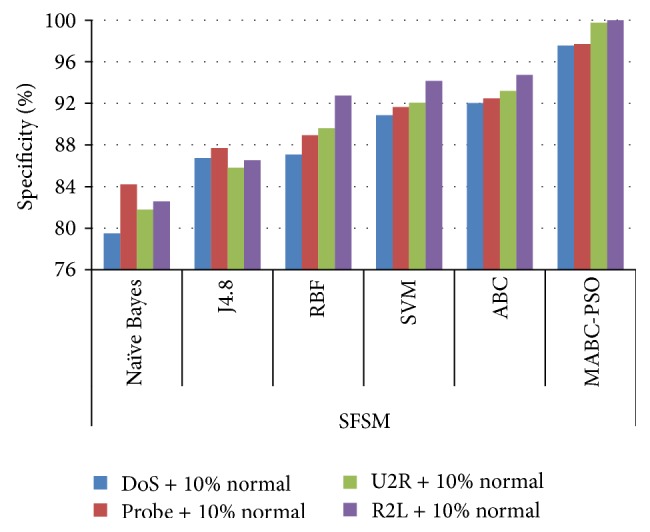
Comparison on specificity using SFSM method.

**Figure 10:**
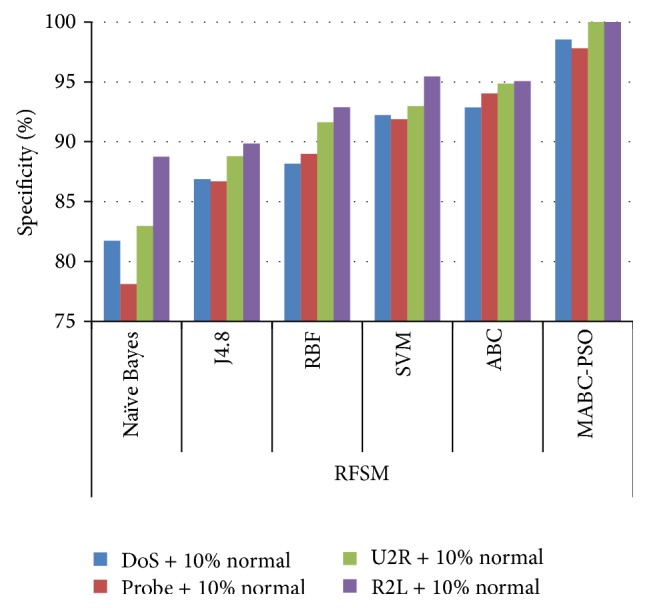
Comparison on specificity using RFSM method.

In Table 12, the results are reported for detection rate obtained by various classification algorithms using different feature selection methods. The comparison results of sensitivity and specificity obtained by proposed method using the two feature selection methods are given in Figures 7 [Fig fig8] [Fig fig9]–10. The results show that on classifying the dataset with all features, detection rate of 87.5%, 83.64%, and 87.16% is obtained for SVM, ABC, and proposed MABC-EPSO approaches. On applying the single feature selection method, detection rate of SVM, ABC, and proposed MABC-EPSO is increased significantly to 88.97%, 89.90%, and 98.09%, respectively. The highest detection rate (98.67%) is reported when the proposed MABC-EPSO with random feature selection method is employed. MABC-EPSO with SFSM also shows a comparable performance than other classifier combinations. The performance of NB, j4.8, and RBF is better in terms of specificity and sensitivity using RFSM method compared to SFSM method.

Table 13 shows the ANOVA results of analyzing the performance of the classifiers based on specificity. In both SFSM and RFSM methods, ANOVA test determined that there are significant differences among the classification algorithms and rejected null hypothesis as calculated *F*(5,18 = 52.535) and *F*(5,18 = 23.539) are greater than* F*-critical (2.773). Finally, multiple comaprison test concluded that MABC-EPSO has significant differences with all the classification algorithms with 0.05 (*P* = 0.05) as significance level. However, there is no statistically significant difference between the SVM and ABC algorithms.

**Figure 11:**
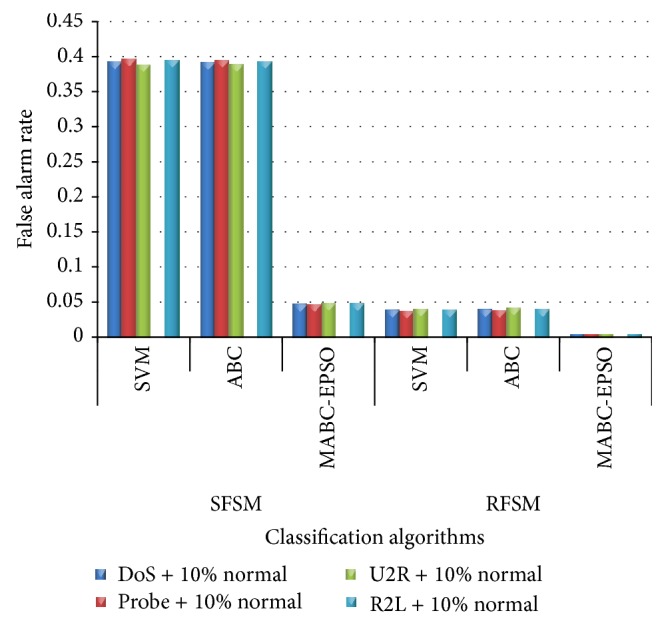
Performance comparison on false alarm rate of classifiers.

**Figure 12:**
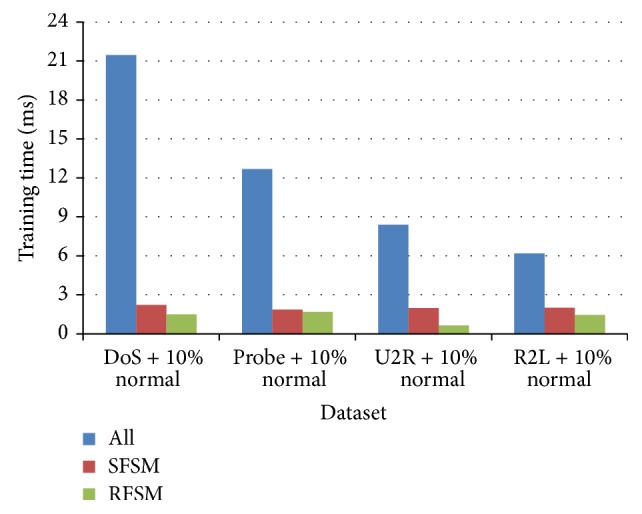
Training time of MABC-EPSO.

Experiment was conducted to analyze the false alarm rate and training time of each classifier using SFSM and RFSM methods.  Figure 11 indicates that MABC-EPSO produces lowest FAR (ranging from 0.004 to 0.005) using RFSM for all datasets. Also, the proposed hybrid approach using SFSM shows a comparable performance with SVM and ABC classifiers using RFSM method.  Table 14 shows that the training time of proposed approach has been significantly reduced for both feature selection methods when compared to other classification algorithms. Training time of the proposed hybrid classifier considering all features is also recorded in Figure 12. The results indicate that the time taken by proposed approach is considerably more when all features are employed. It is also observed that the time consumed by the proposed classifier using the features of RFSM method is comparatively lesser than SFSM method. According to the performance of MABC-EPSO with random feature selection method, the proposed method can be used to solve intrusion detection as classification problem.

## 6. Conclusion

In this work, a hybrid algorithm based on ABC and PSO was proposed to classify the benchmark intrusion detection dataset using the two feature selection methods, SFSM and RFSM. A study of different machine learning algorithms was also presented. Performance comparisons amongst different classifiers were made to understand the effectiveness of the proposed method in terms of various performance metrics. The main goal of this paper was to show that the classifiers were significantly different and the proposed hybrid method outperforms other classifiers. Friedman test and ANOVA test was applied to check whether the classification algorithms were significantly different. Based on the conclusion of ANOVA test, the null hypotheses were rejected, if they were significant. Post hoc analysis using Tukey's test was applied to select which classification algorithm was significantly different from the others. The experiments also showed that the effectiveness of ABC is comparable to the proposed hybrid algorithm. In general, the proposed hybrid classifier produced best results using the features of both SFSM and RFSM methods and is also significantly different from other classification algorithms. Hence, MABC-EPSO can be considered as a preferable method for intrusion detection that outperforms its counterpart methods. In the future, we will further improve feature selection algorithm and investigate the use of bioinspired approaches as classification algorithm in the area of intrusion detection.

## Figures and Tables

**Figure 6:**
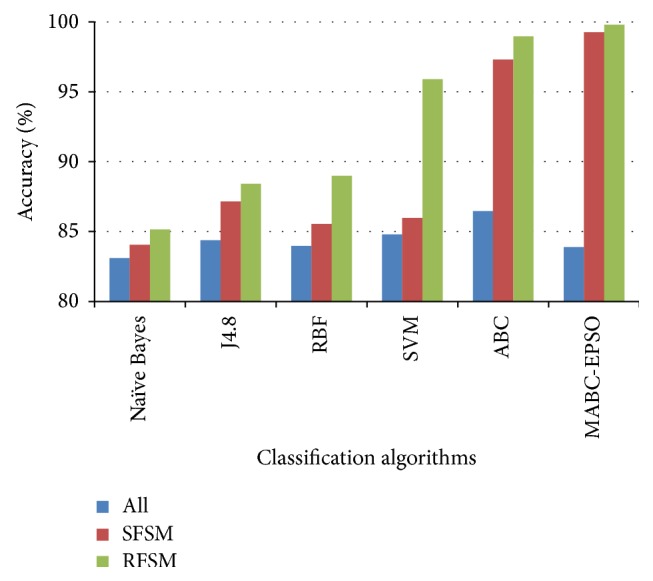
Accuracy comparison of classifiers for U2R dataset.

**Algorithm 1:**
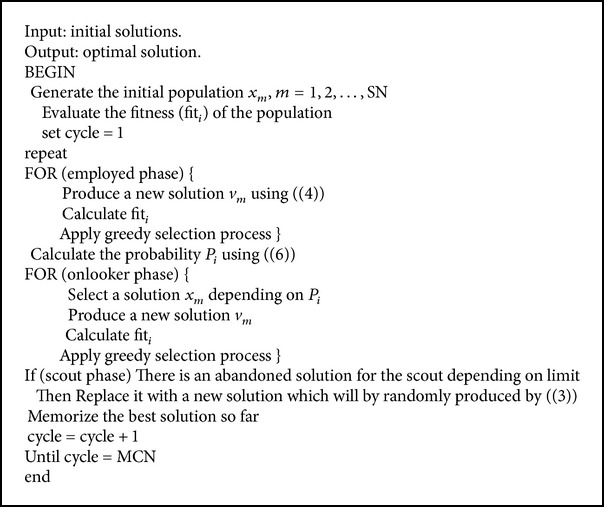
Artificial Bee Colony.

**Algorithm 2:**
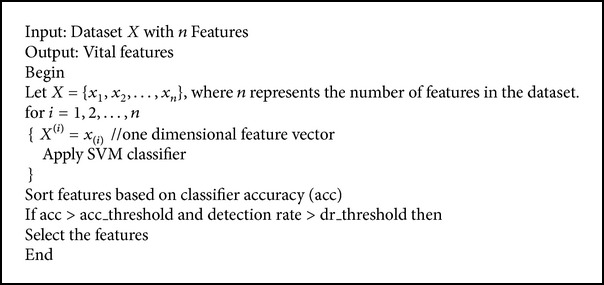
Single feature selection method.

**Algorithm 3:**
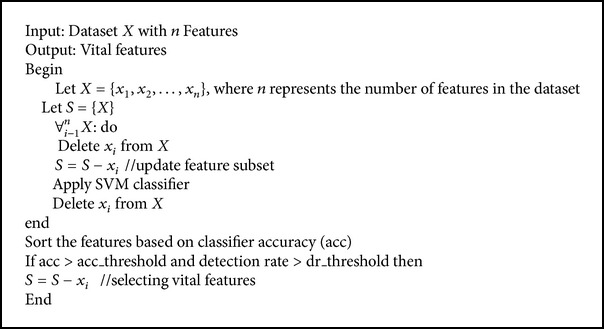
Random feature selection method.

**Table 1:** Distribution of connection types in 10% KDDCup'99 dataset.

Label	% of occurrence					
	DoS	Probe	U2R	R2L	Total attack	Total normal
Training data	79.24%	0.83%	0.01%	0.23%	80.31%	19.69%
Testing data	73.90%	1.34%	0.07%	5.20%	81.51%	19.49%

**Table 2:** Sample size in 10% KDDCUP dataset.

Category of attack	Attack name
Normal	Normal (97277)

DoS	Neptune (107201), Smurf (280790), Pod (264), Teardrop (979), Land (21), Back (2203)

Probe	Portsweep (1040), IPsweep (1247), Nmap (231), Satan (1589)

U2R	Bufferoverflow (30), LoadModule (9), Perl (3), Rootkit (10)

R2L	Guesspassword (53), Ftpwrite (8), Imap (12), Phf (4), Multihop (7), Warezmaster (20), Warezclient (1020)

**Table 3:** Feature information of 10% KDDCUP dataset.

Dataset characteristics	Multivariate
Attribute characteristics	Categorical, integer
Associated task	Classification
Area	Computer
Number of instances	494,020
Number of attributes	42
Number of classes	1 normal class, 4 attack classes

**Table 4:** Details of instances in the dataset.

	Before removing duplicates	After removing duplicates	Selected instances
Normal	97,277	87832	8783
DoS	391,458	54572	7935
Probe	4,107	2131	2131
U2R	52	52	52
R2L	1,126	999	999

Total	494,020	145,586	19,900

**Table 5:** List of features selected using SFSM methods.

Dataset	Selected features	Number of features
DoS + 10% normal	24, 32, 41, 28, 40, 27, 34, 35, 5, 17, 21, 4, 39, 11, 9, 7, 14, 1, 30, 6	20

Probe + 10% normal	11, 1, 15, 26, 10, 4, 21, 18, 19, 25, 39, 31, 7, 35, 28	15

R2L + 10% normal	16, 26, 30, 3, 7, 21, 6, 14, 12, 35, 32, 18, 38, 17, 41, 10, 31	17

U2R + 10% normal	27, 40, 26, 1, 34, 41, 7, 18, 28, 3, 20, 37, 11	13

**Table 6:** List of features selected using RFSM methods.

Dataset	Selected features	Number of features
DoS + 10% normal	4, 9, 21, 39, 14, 28, 3, 8, 29, 33, 17, 12, 38, 31	14

Probe + 10% normal	27, 2, 3, 30, 11, 33, 23, 9, 39, 20, 21, 37	12

R2L + 10% normal	24, 15, 23, 7, 25, 16, 8, 33, 29, 38, 21, 30, 32	13

U2R + 10% normal	6, 19, 22, 30, 21, 28, 36, 27, 11, 17, 20	11

**Table 7:** Confusion matrix.

Actual	Predicted	
	Normal	Attack
Normal	True Negative (TN)	False Positive (FP)
Attack	False Negative (FN)	True Positive (TP)

True Positive (TP): the number of of attacks that are correctly identified.

True Negative (TN): the number of normal records that are correctly classified.

False Positive (FP): the number of normal records incorrectly classified.

False Negative (FN): the number of attacks incorrectly classified.

**Table 8:** Performance comparison of classification algorithms on accuracy rate.

Classification Algorithms	Average accuracy (%)	Feature selection method
C4.5 [6]	99.11	All features
	98.69	Genetic algorithm
	98.84	Best-first
	99.41	Correlation feature selection

BayesNet [6]	99.53	All features
	99.52	Genetic algorithm
	98.91	Best-first
	98.92	Correlation feature selection

ABC-SVM [7]	92.768	Binary ABC
PSO-SVM [7]	83.88	
GA-SVM [7]	80.73	

KNN [8]	98.24	All features
	98.11	Fast feature selection

Bayes Classifier [8]	76.09	All features
	71.94	Fast feature selection

ANN [9]	81.57	Feature reduction

SSO-RF [10, 11]	92.7	SSO

Hybrid SSO [12]	97.67	SSO

RSDT [13]	97.88	Rough set

ID3 [13]	97.665	All features
C4.5 [13]	97.582	

FC-ANN [14]	96.71	All features

**Proposed MABC-EPSO**	88.59	All features
	99.32	Single feature selection method
	**99.82**	Random feature selection method

**Table 9:** Accuracy rates of classifiers using SFSM feature selection method and Friedman ranks.

Dataset	NB	J4.8	RBF	SVM	ABC	MABC-EPSO
DoS + 10% normal	82.57 (6)	87.11 (4)	87.96 (3)	84.7 (5)	90.82 (2)	99.50 (1)
Probe + 10% normal	82.68 (5)	82.6 (6)	83.72 (4)	85.67 (3)	96.58 (2)	99.27 (1)
R2L + 10% normal	86.15 (4)	82.55 (6)	85.16 (5)	90.61 (3)	92.72 (2)	99.24 (1)
U2R + 10% normal	84.06 (6)	87.16 (3)	85.54 (5)	85.97 (4)	97.31 (2)	99.8 (1)

Average rank	5.25	4.75	4.25	3.75	2	1

**Table 10:** Accuracy rates using RFSM feature selection method and Friedman ranks.

Dataset	NB	J4.8	RBF	SVM	ABC	MABC-EPSO
DoS + 10% normal	83.04 (6)	90.05 (4)	88.83 (5)	94.02 (3)	96.43 (2)	99.81 (1)
Probe + 10% normal	84.01 (5)	82.72 (6)	85.94 (4)	95.87 (3)	97.31 (2)	99.86 (1)
R2L + 10% normal	86.32 (4)	83.10 (6)	86.11 (5)	97.04 (3)	98.96 (2)	99.80 (1)
U2R + 10% normal	85.15 (6)	88.42 (5)	88.98 (4)	95.91 (3)	98.96 (2)	99.80 (1)

Average rank	5.25	5.25	4.5	3	2	1

**Table 11:** ANOVA results for accuracy rate of classifiers.

Source of variation	SS	df	MS	*F*	*P* value	*F*-crit.
	SFSM method					
Between groups	781.5143	5	156.3029	31.89498	<0.05	2.772853
Within groups	88.20985	18	4.900547			

Total	869.7241	23				

	RFSM method					
Between groups	879.4307	5	175.8861	48.54728	<0.05	2.772853
Within groups	65.21375	18	3.622986			

Total	944.6444	23				

^*∗*^SS: sum of squared deviations about mean; df: degrees of freedom; MS: variance.

**Table 12:** Performance comparison of classification algorithms on detection rate.

Classification Algorithm	Average detection rate (%)	Feature selection method
Naïve Bayes [15]	92.27	Genetic algorithm
C4.5 [15]	92.1	
Random forest [15]	89.21	
Random tree [15]	88.98	
REP tree [15]	89.11	
Neurotree [15]	98.38	

GMDH Based neural network [16]	93.7	Information gain
	97.5	Gain ratio
	95.3	GMDH

Neural network [17]	81.57	Feature reduction

Hybrid evolutionary neural network [18]	91.51	Genetic algorithm

Improved SVM (PSO + SVM + PCA) [19]	97.75	PCA

Ensemble Bayesian combination [20]	93.35	All features

Voting + j48 + Rule [21]	97.47	All features
Voting + AdaBoost + j48 [21]	97.38	

Rough set neural network algorithm [22]	90	All features

PSO based fuzzy system [23]	93.7	All features

**Proposed MABC-EPSO**	87.16	All features
	98.09	**Single feature selection method**
	**98.67**	**Random feature selection method**

**Table 13:** ANOVA results for specificity of classifiers.

Source of variation	SS	df	MS	*F*	*P* value	*F*-crit.
	SFSM					
Between groups	659.6518	5	131.9304	52.5347	<0.05	2.772853
Within groups	45.20339	18	2.511299			

Total	704.8551	23				

	RFSM					
Between groups	617.818	5	123.5636	23.53957	<0.05	2.772853
Within groups	94.48535	18	5.249186			

Total	712.3033	23				

^*∗*^SS: sum of squared deviations about mean; df: degrees of freedom; MS: variance.

**Table 14:** Training time of classification algorithms using SFSM and RFSM feature selection methods.

Dataset	SFSM						RFSM					
	Naïve Bayes	J4.8	RBF	SVM	ABC	MABC-EPSO	Naïve Bayes	J4.8	RBF	SVM	ABC	MABC-EPSO
DoS + 10% normal	10.20	4.7	3.8	2.86	2.78	2.22	9.95	3.95	3.28	2.59	2.07	1.5
Probe + 10% normal	5.33	3.12	3.05	2.36	2.24	1.87	4.15	3.01	3.19	2.11	1.97	1.69
U2R + 10% normal	4.75	3.81	3.08	2.21	2.16	1.98	4.01	3.46	2.79	1.80	1.78	0.65
R2L + 10% normal	3.98	4.97	3.01	2.46	2.23	2.0	3.12	3.23	2.55	1.42	1.37	1.46
